# Pleiotropic Effects of PCSK-9 Inhibitors

**DOI:** 10.3390/ijms22063144

**Published:** 2021-03-19

**Authors:** Marcin Basiak, Michał Kosowski, Marcin Cyrnek, Łukasz Bułdak, Mateusz Maligłówka, Grzegorz Machnik, Bogusław Okopień

**Affiliations:** Department of Internal Medicine and Clinical Pharmacology, Medical University of Silesia, Medyków 18, 40-752 Katowice, Poland; mbasiak@sum.edu.pl (M.B.); mcyrnek@sum.edu.pl (M.C.); lbuldak@sum.edu.pl (Ł.B.); mmaliglowka@sum.edu.pl (M.M.); gmachnik@sum.edu.pl (G.M.); bokopien@sum.edu.pl (B.O.)

**Keywords:** pleiotropic effects, PCSK-9, anti-atherosclerotic effect, anti-aggregation effect, anticoagulant effect, antineoplastic effect, bacterial infections, vaccine, hyperlipidemia, new lipid lowering-drugs

## Abstract

Proprotein convertase subtilisin/kexin type 9 (PCSK-9) inhibitors are a group of drugs whose main mechanism of action is binding to the PCSK-9 molecule, which reduces the degradation of the low-density lipoprotein receptor (LDL-R) and, hence, increases the uptake of low-density lipoprotein cholesterol (LDLc) from the bloodstream as well as reducing its concentration. The effectiveness of three monoclonal antibodies, namely, alirocumab (human IgG1/κ monoclonal antibody, genetically engineered in Chinese hamster ovary cells), evolocumab (the first fully human monoclonal antibody), and bococizumab (humanized mouse antibody), in inhibiting the action of PCSK-9 and reducing LDLc levels has been confirmed. The first two, after clinical trials, were approved by the Food and Drug Administration (FDA) and are used primarily in the treatment of autosomal familial hypercholesterolemia and in cases of statin intolerance. They are currently used both as monotherapy and in combination with statins and ezetimibe to intensify therapy and achieve therapeutic goals following the American Heart Association (AHA) and European Society of Cardiology (ESC) guidelines. However, the lipid-lowering effect is not the only effect of action described by researchers that PCSK-9 inhibitors have. This paper is a review of the literature describing the pleiotropic effects of PCSK-9 inhibitors, which belong to a group of drugs that are being increasingly used, especially when standard lipid-lowering therapy fails. The article focuses on activities other than lipid-lowering, such as the anti-atherosclerotic effect and stabilization of atherosclerotic plaque, the anti-aggregation effect, the anticoagulant effect, the antineoplastic effect, and the ability to influence the course of bacterial infections. In this publication, we try to systematically review the current scientific data, both from our own scientific work and knowledge from international publications.

## 1. Introduction

In February 2003, Nabil Seidah, a scientist at the Clinical Research Institute of Montreal in Canada, discovered a novel human proprotein convertase, the gene for which was located on the short arm of chromosome 1 [1]. This convertase was called proprotein convertase subtilisin/kexin type 9 (PCSK-9). PCSK-9 is the enzyme responsible for the inhibition of the low-density lipoprotein receptor (LDL-R), which is involved in the metabolism of circulating low-density lipoprotein (LDL) particles. In normal conditions, circulating LDL particles bind to the LDL-R on the surface of hepatocytes. This process causes endocytosis of the complexes to form and then intracellular metabolism of the LDL particle. PCSK-9 binds to the LDL-R located on the cell surface and promotes its lysosomal degradation, thus reducing the amount of available free receptors on the surface of hepatocytes. In the intestines, kidneys, and brain, this process is much less intense [2]. This is the reason why mutations of the PCSK-9 gene significantly affect the concentration of low-density lipoprotein cholesterol (LDLc) in the bloodstream [3,4]. Increased plasma levels of PCSK-9 are observed in patients suffering from familial hypercholesterolemia and during statin therapy. Importantly, research indicates that it is equally dangerous for people suffering from atherogenic dyslipidemia and heterozygous familial hypercholesterolemia independent of LDL-R defects [5]. The PCSK-9 gene also contains one of 27 loci associated with an increased risk of coronary artery disease [6].

**Figure 1:**
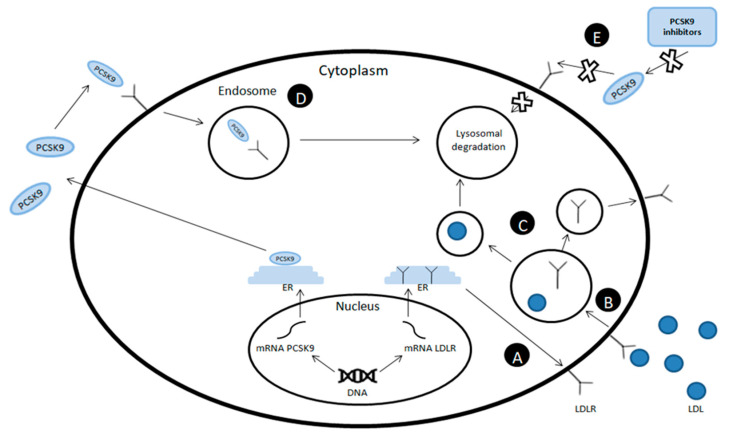
Mechanism of action of proprotein convertase subtilisin/kexin type 9 (PCSK-9) inhibitors. (**A**) Intracellularly produced low-density lipoprotein receptor (LDL-R) is transported to the cell membrane, where it is responsible for binding low-density lipoprotein (LDL) particles. (**B**) After LDL is attached to LDL-R, the complex is endocytosed and then broken down into substrates in the endosome. (**C**) The LDL molecule undergoes lysosomal degradation, and the LDL-R is transported back to the cell membrane. (**D**) At the same time, in other parts of the cell membrane, PCSK-9 binds with LDLR, which causes endocytosis of the complex thus formed and its subsequent degradation in the lysosome. (**E**) The use of PCSK9 inhibitors causes the binding of free PCSK-9 molecules, which prevents them from binding to LDL-R and subsequent receptor degradation. ER: endoplasmic reticulum.

PCSK-9 inhibitors are a group of drugs whose main mechanism of action is binding to the PCSK-9 molecule, preventing it from binding to the LDL-R, reducing the degradation of this receptor, increasing the uptake of LDLc from the bloodstream, and reducing its concentration [7] (Figure 1). In this group, there are three monoclonal antibodies, whose effectiveness in inhibiting the action of PCSK-9 and reducing LDLc levels has been confirmed. They are alirocumab (human IgG1/κ monoclonal antibody, genetically engineered in Chinese hamster ovary cells), evolocumab (fully human monoclonal antibody), and bococizumab (humanized mouse antibody). The first two, after clinical trials, were approved by the Food and Drug Administration (FDA) and are used primarily in the treatment of autosomal familial hypercholesterolemia [8] and in the case of patients with statin intolerance [9]. They are currently used both as monotherapy and in combination with statins and ezetimibe to intensify therapy and achieve therapeutic goals following the American Heart Association (AHA) and European Society of Cardiology (ESC) guidelines [10].

However, the lipid-lowering effect is not the only effect of action described by researchers that PCSK-9 inhibitors have. This study aims to systematize knowledge about other effects of PCSK-9 inhibitors not directly related to the effect on LDL-R and LDLc reduction. This publication is a systematic review of the current scientific data, originating from both our proprietary work and knowledge from international reports. For this purpose, the “PubMed” and “Google Scholar” databases were reviewed in order to isolate reports according to the following key phrases: “PCSK-9 inhibitors inflammation”, PCSK-9 inhibitors pleiotropic effects”, and “PCSK-9 atherothrombosis”.

## 2. Anti-Atherosclerotic Effect and Stabilization of Atherosclerotic Plaque

The main goal of lowering LDLc is to inhibit atherogenesis. Interesting results were obtained in the 52-week OSLER study, which included patients from earlier shorter studies with the following acronyms: MENDEL, LAPLACE-TIMI, GAUSS, and RUTHERFORD. LDLc concentrations in patients on a low cholesterol diet and treated with evolocumab for 52 weeks were reduced by 51.5% from initial values. Studies with another PCSK-9 inhibitor, alirocumab, also show promising results. In one of them, ODYSSEY LONG TERM [11], among patients with heterozygous familial hypercholesterolemia and patients with a high risk of cardiovascular disease (CVD) taking a statin and/or other lipid-lowering drugs with an LDLc concentration not lower than 70 mg/dL, a significant reduction in LDLc was demonstrated in the group receiving alirocumab 150 mg two times a month, by 62% on average compared with placebo over the 24-week follow-up period. The greatest decrease in LDLc concentration was recorded in the fourth week of the study. It is worth noting that as many as 79% of patients achieved the LDLc values recommended by ESC in the group of very high risk of death due to CVD. However, it should be remembered that the formation of atherosclerotic plaques is a considerably complicated process, involving numerous cytokines produced in the vascular endothelium, and one of the most important components of the atherosclerotic process is the inflammatory process taking place in the vascular wall [12]. This hypothesis was verified by a clinical trial in which the concentration of interleukin-1β (Il-1β) was reduced using canakinumab, which resulted in a reduction in the frequency of cardiovascular events in the group of patients taking this drug [13,14].

Several reviews also describe the anti-inflammatory effects of statins, the use of which also positively affects cardiovascular risk [15,16,17]. These studies led to the hypothesis that other drugs used in lipid-lowering therapy, such as PCSK-9 inhibitors, will also have the same pleiotropic effect. However, a meta-analysis from 2018 based on 10 studies showed that despite a significant beneficial effect on lowering LDLc levels, PCSK-9 inhibitors have not reduced the concentration of C-reactive protein (CRP), which is the most basic marker of the ongoing inflammatory process [18]. On the other hand, the effect of PCSK-9 inhibition on concentrations of interleukin-1α (Il-1α), interleukin-6 (Il-6), and tumor necrosis factor α (TNF-α) is completely different. In the conducted studies, in which the activity of PCSK-9 within the atherosclerotic plaque was inhibited with the use of short interfering RNA (siRNA), a significant decrease in the concentrations of the above-mentioned cytokines was observed, which translates into inhibition of the inflammatory process in the plaque [19,20]. Another mechanism described by researchers is increasing the concentration of interleukin-10 (Il-10), known as anti-inflammatory interleukin, by inhibiting PCSK-9. Increasing its concentration reduces the expression of TNFα and the C-C chemokine receptor type 2 (CCR2) [21,22], which is responsible for the influx of monocytes into the atherosclerotic plaque [23]. The last discussed issue is the influence of PCSK-9 on the regulation of the concentration of sirtuins [24], which are a family of proteins involved in histone deacetylation and play a key role in metabolic driver inflammation [25]. To sum up, even though the effect of PCSK-9 inhibition on the reduction of CRP concentration is not visible, it can be seen that this process is very important in inhibiting the ongoing inflammatory process by affecting the expression of key cytokines. Moreover, it also indirectly influences the formation of atherosclerotic plaque through changes in the expression of the CCR2. Currently, however, there are no scientific reports that describe the direct effect of alirocumab and evolocumab on the inflammatory process in the blood vessel wall leading to the enhancement of atherogenesis.

A separate issue is the problem of atherosclerotic plaque destabilization, which is influenced by the expression of PCSK-9 in the vascular endothelium [26]. Studies in which intravascular ultrasound (IVUS) was used suggest that the concentration of PCSK-9 affects the size of the necrotic core within atherosclerotic plaques, regardless of the concentration of LDLc [27]. However, in 2020, there were studies carefully assessing the impact of treatment with PCSK-9 inhibitors on changes in injured atherosclerotic plaques. According to them, although the use of PCSK-9 inhibitors does not affect the size of already formed atherosclerotic plaques, it significantly influences their stabilization by reducing the lipid core burden index [28]. Probably due to this effect, the benefits for patients with diagnosed coronary artery disease using PCSK-9 inhibitors are higher than for patients treated with statins. In our work at the Department of Internal Medicine and Clinical Pharmacology of the Medical University of Silesia, we focused on the problem of the influence of PCSK9 inhibition on the stabilization of atherosclerotic plaque. The first works were presented during the European Atherosclerosis Society and European Society of Hypertension Congresses in 2018. The studies aimed to assess the effect of PCSK9 inhibitors on the serum levels of vulnerable plaque markers in patients with dyslipidemia [29,30]. We chose circulating concentration of soluble ligand CD40 (sCD40L), osteopontin (OPN) and osteoprotegerin (OPG), metalloproteinases (MMPs), and myeloperoxidase (MPO). We concluded that, compared to healthy subjects, dyslipidemic patients exhibited higher baseline levels of all biochemical markers of atherosclerotic plaque progression. PCSK-9 inhibitors decreased the level of all our markers, but this effect did not correlate with their lipid-lowering [29,30].

Subsequent studies focused on the effects of PCSK9 inhibition on biomarkers of atherosclerotic plaque destabilization release in hypertensive patients with dyslipidemia [31,32]. Additionally, we used both serum biomarkers and advanced magnetic resonance techniques. Lipid profile and biomarkers level were determined at the beginning of the study and at 90 days of treatment. In the case of the ruptured plaque suspicion, we decided to perform carotid magnetic resonance imaging (MRI) and analyze the structure of atherosclerotic lesions. Based on the predominant components of the plaque, plaques were characterized as lipid, a lipid with recent hemorrhage, fibrous, fibrofatty, and fibrofatty with some hemorrhagic components. Moreover, we observed increased concentration of osteopontin, osteoprotegerin, metalloproteinase-9, and a positive correlation with sCD40l. Based on preliminary data, it can be concluded that diagnostic imaging methods together with biochemistry markers can provide complete information about the plaque characteristics in hypertensive or dyslipidemic patients [31,32]. The treatment-induced reduction in the release of cytokines and plaque destabilization markers may contribute to the clinical effectiveness of PCSK-9 inhibitors in the therapy of atherosclerosis. These interesting data require further studies in a larger population of patients.

## 3. Anti-Aggregation and Anticoagulant Effects

The topic of lipid-lowering treatment has long been of considerable interest to researchers due the fact that the use of lipid-lowering drugs such as statins has a cardiovascular protective effect that is not directly related to lowering LDLc levels [33]. As shown in some clinical trials, e.g., JUPITER, the reduction of cardiovascular events during the use of statins is significantly greater than that resulting from the reduction of lipid levels [34]. A similar situation applies to PCSK-9 inhibitors. Originally, their potent effect in reducing the number of cardiovascular events was attributed to their LDL-lowering effect [35]. However, this view was changed by a recent meta-analysis in which it was shown on a group of 300,000 patients that the reduction of cardiovascular events is greater in patients using PCSK-9 inhibitors compared to patients using statins with the same level of LDLc reduction [36]. The reasons for such observation are primarily sought in the action of PCSK-9 inhibitors by which they increase high-density lipoprotein (HDL) concentrations and reduce lipoprotein (a) (Lp(a)) concentrations [37].

However, this is not the only mechanism by which these drugs prevent the cardiovascular system. One such mechanism is the mechanism of thrombosis caused by inflammation in the vascular endothelium, in which the CD36 protein and the lectin-like oxidized low-density lipoprotein receptor-1 (LOX-1) on the surface of platelets are involved [38,39]. CD36 is an LDL and ox-LDL binding protein that plays a key role in the formation of blood clots [40], while LOX-1 is a receptor whose expression is triggered by inflammatory stimuli [41]. Another mechanism by which dyslipidemia may affect platelets is the Toll-like receptor 2 (TLR2) stimulation mechanism, which activates the aggregation process through lipid-peroxide-modified phospholipids in the transport of Lp(a), which plays an important role [42,43]. Taking into account the mechanisms discussed above, inhibition of PCSK-9 may contribute to the reduction of platelet activity at several levels. First, by lowering the plasma concentration of LDLc, they can lower the level of cholesterol in the plasma membrane of platelets and thus reduce their activity. Secondly, given the positive effect of PCSK-9 on LOX-1 expression, PCSK inhibitors can reduce the number of LOX-1 [44]. Additionally, by reducing Lp(a) levels, which distinguishes them from statins, they inhibit platelet activation via peroxide-modified phospholipids [45]. Moreover, a decrease in platelet activity results in a decrease in oxidized low-density lipoprotein (oxLDL) synthesis, and, thus, it breaks the vicious cycle promoting thrombus formation [46]. Bearing in mind the results of the above-mentioned research, a clinical trial was conducted, proving that treatment with alirocumab and evolocumab reduces the activation of platelets [47]. It should be noted, however, that the formation of an embolus is caused not only by the aggregation of platelets. The coagulation factor system is also involved in this process. Epidemiological studies have shown that in patients after ischemic stroke or myocardial infarction, the frequency of relapses correlated with increased levels of factor VIII (FVIII) [48,49], but in the group of patients diagnosed with thrombophilia, the incidence of ischemic heart disease was significantly lower than in the general population [50]. The plasma concentration of FVIII is regulated by its biosynthesis and purification via LDL-R and low-density lipoprotein receptor-related protein 1 (LPR1). Most of the circulating FVIII that binds to the von Willebrand factor (VWF) does not bind to receptors on hepatocytes. However, the unbound VWF FVIII (accounting for approximately 5% of total circulating FVIII) is rapidly recognized and bound to LPR1, and then endocytosed and degraded in the liver cell [51]. Studies conducted on animal models have shown that the cooperation between LPR1 and LDL-R receptors is also important in this process [52], which is reflected in studies reporting an increased cardiovascular risk in a population with diagnosed LDL-R gene polymorphism, which was also associated with an increased concentration of FVIII in this population [53]. Randomized studies show that in the group of patients taking high doses of statins, a significant decrease in the plasma concentration of FVIII was observed [54], which is related to the influence of this group of drugs on stimulating the expression of LDL-R and LPR1 [55,56]. Currently, despite the mechanism of action being similar to that of statins, there are no clear reports on the effect of PCSK-9 inhibitors on the plasma concentration of FVIII.

In connection with the above-described mechanism and anti-inflammatory and anti-atherosclerotic effects of PCSK-9 inhibitors, the impact of their use on the incidence of venous thromboembolism is also considered, as it is known that the ongoing inflammation and atherosclerotic process are associated with the occurrence of this disease [57,58]. Although studies do not show any correlation between LDLc concentration and the occurrence of venous thromboembolism [59], the relationship between Lp(a) concentration and the risk of this disease is completely different [60]. This is important because the statins used to date in lipid-lowering therapy lower the concentration of LDLc and triglycerides but do not affect the concentration of Lp(a). PCSK-9 inhibitors have a different effect, reducing both the concentration of LDLc and Lp(a) [61]. In connection with the above reports, a study was conducted to assess the effect of alirocumab on the incidence of venous thromboembolism, which showed that alirocumab significantly reduces the risk of venous thromboembolism when compared to the group of patients using the placebo, which was associated with a significant decrease in Lp(a) levels after drug administration [62].

## 4. PCSK-9 Inhibitors and Bacterial Infections

An interesting topic is the relationship between lipid-lowering treatment and the tendency for the occurrence of bacterial infections and accompanying life-threatening condition, such as sepsis and septic shock. It is known that the Toll-like receptor (TLR) plays a key role in initiating the immune response against pathogens attacking our body, and the blockade of this receptor has a protective effect and delays the occurrence of septic shock in response to infection [63]. This receptor is naturally activated by lipid molecules associated with the cell walls, which include lipopolysaccharides (LPS) in the case of G (−) bacteria, lipoteichoic acid in the case of G (+) bacteria, and finally phospholipomannan in the case of fungi. The concentration of these lipids is proportional to the strength of the immune system response and, thus, the severity of the infection [64]. It is also known that these lipids in the bloodstream are incorporated into HDL particles and then transferred to LDL and very low-density lipoproteins (VLDL), which are captured by the LDL-R and thus eliminated from the bloodstream. This is why the key role in the rapid elimination of lipids that can accelerate the development of septic shock is played by PCSK-9 molecules responsible for the inhibition of the LDL-R, which was proven in the study by comparing pro-inflammatory cytokine levels after LPS exposure in people with a mutation causing loss of PCSK-9 function with groups of people with a mutation that enhances the activity of PCSK-9. This study proved a significant reduction in the concentration of pro-inflammatory cytokines in the group of people with a mutation causing loss of PCSK-9 function [65].

In the experimental sepsis model, PCSK-9 gene overexpressing transgenic mice subjected to cecal ligation and puncture were characterized by a much greater increase in IL-6, thrombin–antithrombin (TAT), and the incidence of kidney and liver damage compared to the control group. Moreover, in a similar model of sepsis carried out in mice with decreased expression of the gene for PCSK-9, a decrease in the concentration of myeloperoxidase and IL-10 and a reduction in the frequency of lung and liver damage were characteristic [66]. These studies show that PCSK-9 stimulates an excessive inflammatory response, which may contribute to the rapid progression of sepsis to septic shock. Apart from the proven influence on the concentration of pro-inflammatory cytokines, one should not forget about the stimulating effect of PCSK-9 on the activation and proliferation of T lymphocytes [67], proven in vitro, which also plays a role in the development of this disease. Taking these data into account, it can be concluded that the use of drugs aimed at inhibiting the function of PCSK-9 should improve the prognosis of patients who develop sepsis as a result of infection. Currently, however, there is no scientific evidence unambiguously confirming this thesis. In addition to the above-mentioned evidence on the important role that PCSK-9 inhibitors may play in slowing down the progression of bacterial and fungal infections, a single report also mentioned the likely positive effect of the lipid-lowering effect of PCSK-9 inhibitors in the prevention and treatment of malaria parasite infection [68].

## 5. Antineoplastic Effect

Due to many scientific reports dealing with the negative impact of increased levels of LDLc and triglycerides on the risk of developing colorectal cancer [69,70], research has started to determine the effect of mutations within the gene for PCSK-9 on the risk of cancer development. Studies on the risk of breast cancer in correlation with the concentration of lipids in the bloodstream showed that mutations causing a decrease in PCSK-9 activity and, thus, a decrease in LDLc concentration, were associated with a lower risk of this type of cancer [71]. Additionally, in vitro studies on lung adenocarcinoma and glioma cell lines showed the effect of PCSK-9 inhibition on the apoptosis of neoplastic cells [72,73]. Therefore, studies have been carried out in animal models to determine the effect of administering a vaccine to stimulate the immune system to produce antibodies against the PCSK-9 molecule on the development of certain types of cancer. These studies have proven that inhibition of PCSK-9 is associated with inhibition of the progression of colorectal cancer and breast cancer [74,75]. To date, however, there are no clinical trials that describe the effect of the use of PCSK-9 inhibitors used in lipid-lowering therapy on the development and progression of neoplasms in cancer patients.

## 6. Virus-Like Particle (VLP) Vaccine

A vaccine against PCSK-9 has been developed for the treatment of patients with very high levels of LDLc. The vaccine uses a VLP as the immunogenic carrier of the antigenic peptide PCSK-9. VLPs are viruses that have had their DNA removed so that they retain their external structure and their ability to antigen presentation. Although they cannot replicate, they can elicit an immune response without causing infection. Mice and macaques vaccinated with VLP bacteriophages, which present peptides derived from PCSK-9, produced high-titer IgG antibodies, which then bound to circulating PCSK-9. Vaccination was associated with significant reductions in total cholesterol, free cholesterol, phospholipids, and triglycerides [76]. New experimental data should also be mentioned. Yajie Pan et al. found that mice vaccinated with VLP-PCSK9 peptide vaccines, especially the PCSK9Qβ-003 vaccine, developed high-titer IgG antibodies against PCSK9. The PCSK9Qβ-003 vaccine clearly decreased plasma total cholesterol plasma PCSK9 levels and also up-regulated LDLR expression in the liver. Additionally, vaccine injection was associated with significant up-regulation of sterol-regulatory element-binding protein-2 (SREBP-2), hepatocyte nuclear factor 1α (HNF-1α), and 3-hydroxy-3-methylglutaryl coenzyme A (HMG-CoA) reductase [77]. In another study, Danyu Wu et al. demonstrated that the PCSK9Qβ-003 vaccine attenuated the progression of atherosclerosis by modulating reverse cholesterol transport and inhibiting inflammation infiltration and apoptosis, which may provide a novel therapeutic approach for atherosclerosis and greatly improve treatment compliance among patients [78]. Currently, studies are being prepared that could also be methodologically carried out in humans.

## 7. Safety of PCSK-9 Inhibitors

An unquestionable advantage of PCSK-9 inhibitors is their better tolerance compared to the lipid-lowering drugs used to date. They cause significantly fewer muscle symptoms than statins [9,79]. As these agents are increasingly used in current clinical practice, mounting scientific and clinical evidence supports that PCSK9 inhibitors offer an excellent safety and tolerability profile with a low incidence of adverse events. In the FOURIER trial from 2017, the percentage of reported adverse events after administration of evolocumab was significantly lower compared to previous studies, and more importantly, the number of adverse events did not differ significantly between the study group and the placebo group [80]. Detailed data are presented in Table 1.

Moreover, an FDA warning in March 2014 about possible cognitive adverse effects of PCSK-9 inhibition caused concern, as the FDA asked companies to include neurocognitive testing into their phase III clinical trials. In 2020 Gencer et al. concluded that the addition of evolocumab to maximally tolerated statin therapy had no impact on patient-reported cognition after an average of 2.2 years of treatment, even among patients who achieved LDL-C < 20 mg/dL [81]. The occurrence of ADAs (anti-drug antibodies) with PCSK9 inhibitors has attracted widespread attention. Although autoantibodies were detected in some patients on alirocumab or evolocumab, reductions in LDL-C levels induced by either monoclonal antibody were not attenuated [82]. PCSK9 inhibitors do not appear to exert a detrimental effect on glycemic control or to increase the incidence of new-onset diabetes mellitus. Accumulating evidence also indicates that PCSK9 inhibitors are a safe, well-tolerated and effective therapeutic strategy for patients with statin intolerance. On the other hand, as PCSK9 inhibitors reduce LDL-C to unprecedented low levels, a large body of current research has examined the effects of their long-term administration on neurocognition and on levels of vitamin E and other fat-soluble vitamins, providing encouraging results [80,83,84].

## 8. Conclusions

PCSK-9 inhibitors are a group of drugs that are increasingly used in patients who receive intensive lipid-lowering therapy to reduce the risk of cardiovascular events. It can be seen, however, that many of the mechanisms of action by which these drugs reduce cardiovascular risk in patients are not yet known and require further research. On the other hand, many of the already-known mechanisms of action described in animal or cell models still require confirmation in clinical trials. However, this does not change the fact that the beneficial effect of their use is unquestionable, which prompts an increasing number of physicians to use this group of drugs both in the primary and secondary prevention of cardiovascular incidents.

## Figures and Tables

**Table 1:** Adverse events following the administration of evolocumab versus placebo [80].

Outcome	Evolocumab (%)	Placebo (%)
Injection-site reaction	2.1	1.6
Allergic reaction	3.1	2.9
Muscle-related event	5	4.8
Rhabdomyolysis	0.1	0.1
Cataract	1.7	1.8
Adjudicated case of new-onset diabetes	8.1	7.7
Neurocognitive event	1.6	1.5
Aminotransferase level >3 times the upper limit of the range	1.8	1.8
Creatine kinase level >3 times the upper limit of the range	0.7	0.7

## Data Availability

Not applicable.
